# Intratumoral and peritumoral habitat radiomics of MRI predicts pathologic complete response to neoadjuvant chemoimmunotherapy in oral squamous cell carcinoma

**DOI:** 10.1097/JS9.0000000000002715

**Published:** 2025-06-20

**Authors:** Zilong Yuan, Shuangquan Ai, Qian He, Kun Wu, Miao Yang, Kaiyi Zheng, Yaoyao He, Xiaojuan Tang, Yulin Liu, Zheng Wu, Yuan Wu

**Affiliations:** a Department of Radiology, Hubei Cancer Hospital, Tongji Medical College, Huazhong University of Science and Technology, Wuhan. Hubei, China; b Department of Radiation Oncology, The Affiliated Cancer Hospital of Xiangya School of Medicine, Central South University & Hunan Cancer Hospital, Changsha, Hunan, China; c Department of Radiation and Oncology, Hubei Cancer Hospital, Tongji Medical College, Huazhong University of Science and Technology, Wuhan. Hubei, China; d College of Biomedical Engineering, South-Central Minzu University,Wuhan, Hubei, China,; e Department of Radiologic Center, Renmin Hospital, Hubei University of Medicine, Shiyan, Hubei, China

**Keywords:** habitat imaging, intratumoral and peritumoral, oral squamous cell carcinoma, pathologic complete response, radiomics

## Abstract

**Background:**

Neoadjuvant chemoimmunotherapy (NACI) shows promise in oral squamous cell carcinoma (OSCC), but reliable noninvasive biomarkers for predicting pathologic complete response (pCR) remain scarce. Radiomics integrating intratumoral and peritumoral heterogeneity across multi-sequence MRI may offer novel insights into treatment response evaluation.

**Methods:**

The data of 212 patients with OSCC after NACI were retrospectively collected and analyzed. Among these patients, 56 (26.4%) achieved pCR after NACI. Intratumoral and peritumoral habitat imaging (HI) was achieved using the K-means clustering algorithm applied to T1-weighted imaging (T1WI), axial T2-weighted imaging with fat suppression (T2WI), and contrast-enhanced T1-weighted imaging with fat suppression (T1C). Moreover, intratumoral and peritumoral HI models were constructed and compared using the receiver operating characteristic curve (ROC). Five-fold cross-validation was performed to mitigate model overfitting.

**Results:**

Intratumoral HI models derived from different sequences and the Intratumoral Fusion model exhibited favorable predictive ability, with AUCs of 0.738-0.817 and 0.729-0.789 in the training and testing cohorts, respectively. Moreover, peritumoral HI models displayed marginally higher predictive abilities compared to intratumoral HI and Fusion models, with AUCs of 0.734-0.869 and 0.788-0.802 in the training and testing cohorts, respectively. Meanwhile, the decision model with peritumoral habitat features (PHF_S_), intratumoral habitat features (IHF_S_), and 3 clinical features displayed the highest performance, with average AUCs of 0.913 and 0.843 in both respective cohorts. Among the most important features screened by SHAP, three IHFs and one PHF could effectively distinguish between the lower and higher groups of programmed cell death ligand 1 (PD-L1) Combined Positive Score (t = 2.027-2.275, *P* < 0.05), whilst two PHFs were highly correlated with CD45+ white blood cell densities in the stroma (r = 0.958, −0.920, *P* < 0.05), which were associated with pCR.

**Conclusions:**

Integrated intratumoral and peritumoral HI derived from multi-sequence MRI offers a high predictive capacity for pCR following NACI in OSCC patients.

## Introduction

As is well documented, oral squamous cell carcinoma (OSCC) is a relatively prevalent malignant tumor among head and neck squamous cell carcinoma (HNSCC)[1] and is characterized by a poor prognosis. To date, its treatment is largely similar to that of HNSCC. The predominant treatment strategy for HNSCC patients is a comprehensive regimen integrating surgery, radiotherapy, chemotherapy, and targeted therapy. However, a substantial proportion of patients, approximately 40%-60%, ultimately experience local recurrence or distant metastasis, with a 5-year survival rate below 50%[2]. In recent years, immunotherapy, especially immune checkpoint inhibitors (ICIs), has emerged as a promising approach for the treatment of OSCC. Although neoadjuvant chemoimmunotherapy (NACI) can significantly enhance the pathologic complete response (pCR) rate in patients with locally advanced HNSCC, including those with OSCC, roughly 45% of patients fail to achieve pCR[3]. Consequently, there is an urgent and unmet need for the early prediction of NACI efficacy in OSCC patients.

Radiomics, a method for quantifying intratumoral heterogeneity (ITH), has been extensively utilized in diverse aspects of HNSCC^[4-14]^. In the specific subfield of OSCC, earlier studies have also focused on its value in predicting staging[15], cervical lymph node metastasis^[16-20]^, local recurrence[21], bone invasion[22], and overall survival (OS)[23].Regarding the prediction of treatment response in OSCC after NACI, only Liu *et al*[14] attempt to predict post-treatment pCR in HNSCC patients by extracting histogram-related features within tumors from multi-sequence MRI. Nonetheless, it is worth acknowledging that their sample size was limited. Similarly, Lin *et al*[12] developed a radiomics model based on multi-sequence MRI to predict post-treatment pCR in HNSCC patients, albeit using the traditional radiomics approach. Traditional radiomics typically assumes a homogeneous distribution of features within the region of interest (ROI), thereby overlooking intratumoral regional disparities and facing challenges in capturing the complex spatial distribution information within tumors.

In contrast, sub-region-based habitat imaging (HI) principally combines quantitative image analysis with the underlying tumor pathophysiology. Constructing a “habitat map” of the tumor may reveal internal heterogeneity, thereby mirroring tumor growth, drug resistance, pathological grade, and prognosis^[24-28]^. Moreover, given that it can more effectively quantify sub-regions that are more relevant to tumor growth or invasiveness, it is superior to a simple whole-tumor analysis. Concurrently, numerous studies have explored the application of peritumoral radiomics features to characterize the microenvironment for predicting patient outcomes^[29,30]^. However, these efforts have been predominantly limited to the overall analysis of the peritumoral region, with several studies demonstrating that peritumoral HI can also contribute to enhancing the predictive capacity of the model^[31-33]^.

To the best of our knowledge, no studies have employed intratumoral and peritumoral HI to predict pCR following NACI in OSCC. Therefore, this study aimed to establish intratumoral and peritumoral HI models based on multi-sequence MRI to predict the pCR of OSCC patients after NACI. In addition, an interpretability analysis method was introduced to enhance the biological interpretability of the model. To ensure transparency and reproducibility in radiomics analysis, this study adheres to the Transparency In The Reporting of Artificial Intelligence (TITAN) guideline[34] .

## Methods

### Patients

**Figure 1.**
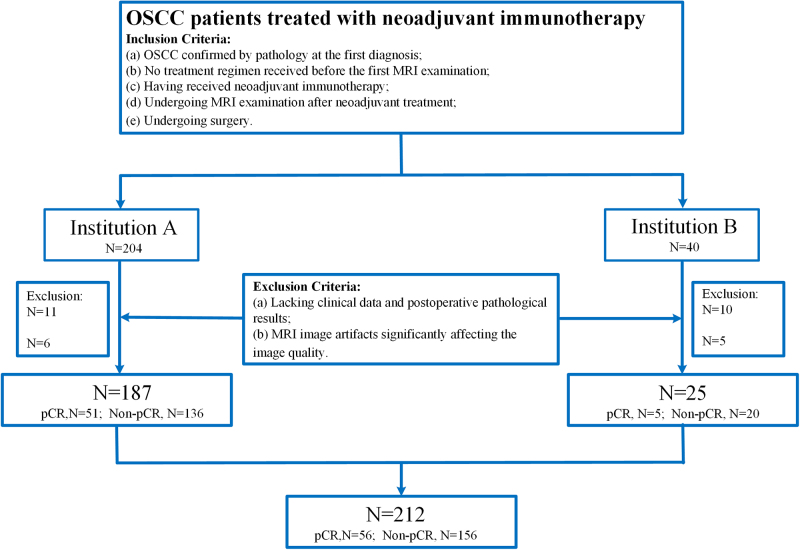
Flowchart of patient inclusion.

This retrospective study was approved by the institutional review board (IRB) of Hunan Cancer Hospital (A) and Hubei Cancer Hospital (B). Due to the retrospective design and use of de-identified data, individual patient consent was waived by the IRB. All patient data, including clinical and imaging information, were anonymized before analysis, with personal identifiers removed and sensitive data encrypted. This study comprehensively reviewed the clinical information of OSCC patients who underwent NACI at two institutions from March 2020 to December 2023. Initially, a total of 244 patients, consisting of 204 patients from Institution A and 40 from Institution B, were identified. Participants were eligible for inclusion if they met the following criteria: (a) histopathologically confirmed OSCC at the initial diagnosis; (b) no treatment received prior to the first MRI examination; (c) receipt of NACI; (d) underwent MRI examination after NACI; (e) Underwent surgical intervention. The exclusion criteria were as follows: (a) Absence of clinical data and postoperative pathological results; (b) MRI image artifacts significantly affecting image quality. Ultimately, the dataset comprised 187 patients from Institute A and 25 patients from Institute B. Among them, 56 achieved pCR, whereas the remaining 156 patients were classified as non-pCR (Fig. 1). This study had been reported in line with the REMARK criteria[35].

**HIGHTLIGHTS**

- In a retrospective analysis of 212 OSCC patients undergoing neoadjuvant chemoimmunotherapy, multi-sequence MRI-derived intratumoral and peritumoral habitat imaging demonstrated robust predictive value for pCR.
- The integrated decision model combining peritumoral habitat features (PHFs), intratumoral habitat features (IHFs), and clinical characteristics achieved superior predictive performance (AUC 0.913/0.843 in training/testing cohorts), outperforming single-sequence or single-region models.
- SHAP-based visualization identified critical habitat features were associated with PD-L1 CPS and CD45+ in the stroma which was correlated with pCR, providing biological interpretability for personalized treatment optimization.

For each patient, baseline characteristics, such as gender, age, smoking history, drinking history, diagnosis of tumor occurrence, pre-treatment TNM staging, baseline hematological index, HPV status, and Programmed Death-Ligand 1 (PD-L1) Combined Positive Score (CPS) were collected from medical records. The number of immunotherapy cycles and TNM staging after neoadjuvant therapy were also collected for analysis. It should be noted that only 33 patients underwent PD-L1 CPS assessment.

### MR examination

In the present study, all patients underwent head and neck MRI scanning. Specifically, Institution A employed the Siemens 1.5 T MRI scanner (Aera), the General Electric (GE) 1.5 T MRI scanner (optima MR360), and the United Imaging 3 T MRI scanner (uMR780), while Institution B utilized the GE 1.5 T MRI scanner (Signa HDXT) and the United Imaging 3 T MRI scanner (uMR790). Subsequently, axial T1-weighted imaging (T1WI), axial fat-suppressed T2-weighted imaging (T2WI), and axial contrast-enhanced T1-weighted imaging with fat suppression(T1C) were acquired for all patients. These highly standardized imaging sequences play a crucial role in the MRI staging process of OSCC. Among them, T1WI provides excellent anatomical detail, T2WI highlights tissue heterogeneity, and T1C emphasizes tumor angiogenesis. Detailed imaging parameters are presented in Supplemental Digital Content, Table S1 (available at: http://links.lww.com/JS9/E435).

### Intratumoral and peritumoral region generation

**Figure 2.**
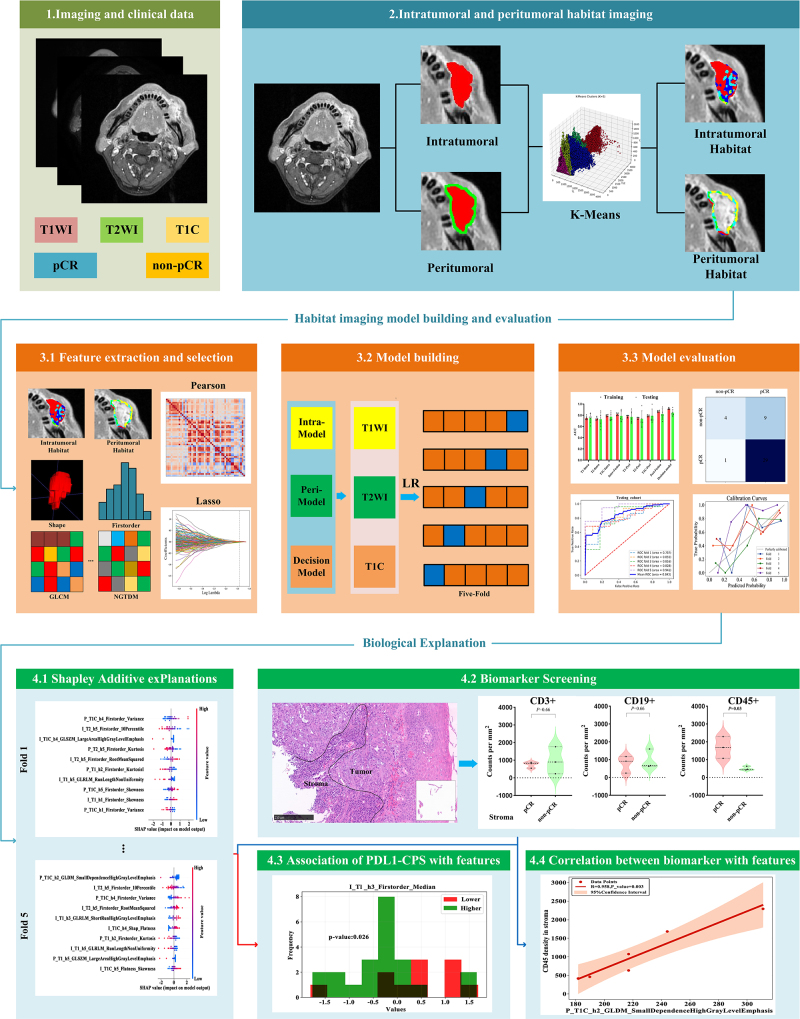
Workflow illustrating radiomics analysis.

Referencing the corresponding T1WI and T2WI images, ROI encompassing the whole tumor across all layers was delineated on T1C images by a radiologist with 10 years of experience. In order to ensure the accuracy of the contour, the contoured area was reviewed by another radiologist with 15 years of experience. Regions of necrosis, hemorrhage, or edema were excluded from the ROIs. Delineation was performed using open-source Slicer software (available at: http://www.slicer.org). Next, the Onekey v.2.2.3platform (available at: http://medai.icu/) was utilized to expand the tumor boundary outward by 3 mm, which was defined as the peritumoral region[12]. The radiologist with 15 years of experience reviewed the peritumoral region to ensure that the expanded regions excluded surrounding cavities and bone tissues, thereby improving the accuracy and reliability of peritumoral segmentation. The workflow for radiomics analysis is illustrated in Fig. 2.

### Image preprocessing

To minimize the impact of images from different imaging devices on the generalizability of radiomics models, this study conducted a two-step post-processing approach. First, the N4 bias field correction method based on the SimpleITK toolkit in Python 3.7.1 was initially employed to eliminate the influence of uneven signal intensity caused by inhomogeneous magnetic field variations across images. Subsequently, the zoom toolkit was utilized to resample these images from three sequences, adjusting the voxel size to 1 mm ×1 mm ×1 mm. Trilinear interpolation was adopted for image resampling, while the nearest neighbor interpolation technique was applied to the masks. Finally, the T1WI and T2WI images were registered onto the T1C images, thus providing an accurate and standardized image dataset for subsequent image analysis.

### Habitat imaging and radiomics feature extraction

Intratumoral and peritumoral habitat regions were primarily defined through the utilization of the sklearn.cluster. Kmeans toolkit in Python 3.7.1. Compared to the Gaussian Mixture Model and the OTSU Method, the K-means clustering method boasts several advantages, including high computational efficiency, robust adaptability to data distribution, and enhanced interpretability of results^[36-38]^. In this study, HI was conducted on T1WI, T2WI, and T1C images. Each voxel within the intratumoral and peritumoral areas encompassed paired data of these three sequences. The combination of these paired data from all voxels across the entire patient cohort generated a data array with a dimension of N × 3, where N denotes the number of image voxels, and 3 corresponds to the parameters of the three sequence images. Thereafter, a K-means clustering method was constructed based on the aforementioned dataset to allocate each voxel to a specific cluster or habitat. K-means clustering is an iterative approach that partitions n data points into k clusters by assigning each data point to the cluster with the closest mean. This method initiates with a random assignment, recalculates the means, and iterates until the cluster assignments stabilize or a predefined limit is attained. The Sum of Squared Errors (SSE) was employed to assess clustering quality. SSE serves as a crucial evaluation metric in the K-means clustering algorithm, estimating the sum of the squared distances from each data point to the center of its affiliated cluster. The elbow method was utilized to determine the optimal value of k by identifying the inflection point of the SSE, where the rate of decline abruptly slows. In this study, the inflection points for both the intratumoral and peritumoral regions were 5, thus subdividing the sub-regions into h1, h2, h3, h4, and h5. Meanwhile, we also performed K-means clustering analysis on data from both centers so as to ensure the robustness of the K-means clustering algorithm across different cohorts, and found that the optimal number of clusters was consistently 5 for both datasets (Supplemental Digital Content, Figure S1, available at: http://links.lww.com/JS9/E434).

In order to avoid overfitting as much as possible, 107 low-dimensional radiomics features were extracted from each sub-region using the Pyradiomics package of Python (version 3.7.1), including the shape (n = 14), first order (n = 18), gray-level co-occurrence matrix (GLCM, n = 24), gray-level run-length matrix (GLRLM, n = 16), gray-level size zone matrix (GLSZM, n = 16), neighborhood gray-tone difference matrix (NGTDM, n = 5), and gray-level dependence matrix (GLDM, n = 14). Finally, 1070 radiomics features were acquired from each sequence for subsequent model construction and analysis.

### Feature selection and model construction

This study employed a two-stage feature selection pipeline to optimize model performance. First, radiomics features were standardized using the Z-score approach to ensure that the mean value of the features was 0 and the variance was 1.Dimensionality reduction was then performed using Pearson correlation coefficient. When the correlation coefficient |R| between two features exceeded 0.95, one of the features was eliminated. The remaining features underwent further refinement, which was performed using the 10-fold cross-validated LassoCV method to identify the most relevant ones for the model.

In this study, five machine learning algorithms, including Logistic Regression (LR), Support Vector Machine (SVM), K-Nearest Neighbors (KNN), Decision Tree (DT), and Random Forest (RF), were used to construct predictive models. To evaluate the prediction models’ validity, five-fold cross-validation was also performed on the dataset. In comparison to the bootstrapping method, five-fold cross-validation is less susceptible to the randomness of data partitioning, yields relatively consistent evaluation outcomes, and thus effectively mitigates the risk of overfitting[39]. Meanwhile, model performance was evaluated using average area under the curve (AUC)value of the receiver operating characteristic (ROC) curve across folds. This approach minimized overfitting and ensured robust evaluation.

This study developed five pCR prediction models: Intratumoral HI models, Intratumoral Fusion model, Peritumoral HI models, Peritumoral Fusion model, and the decision model. The Intratumoral and Peritumoral HI models were constructed using HI features from different sequences, while their respective Fusion models integrated HI features from three sequences. The decision model incorporated clinical characteristics along with both intratumoral and peritumoral HI features.

### Biological interpretability analysis

In order to evaluate the contribution of the finally screened features to the predictive model, SHapley Additive exPlanations (SHAP) analysis was performed to evaluate the interpretability of the linear model, revealing the complex mechanism of machine learning model prediction. SHAP visualizes the contribution of each feature to the model prediction by assigning an importance value to each feature in each prediction, indicating how it increases or decreases the probability of a specific outcome. The SHAP analysis was carried out using the explainers. Linear function of the SHAP toolkit in Python (version 3.7.1).

To further enhance model interpretability, the association between the important radiomic features after SHAP screening in the decision model and PD-L1CPS was comprehensively explored. Given the correlation between PD-L1CPSand treatment effectiveness in head and neck carcinoma, a threshold of CPS greater than or equal to 15 was applied for subsequent analysis. Meanwhile, CD45+ white blood cells, CD3+ T cells, and CD19+ B cells in baseline biopsy tissue were quantified using multiplex immunofluorescence assays in pCR patients (n = 3) and non-pCR patients (n = 3) from Institution B to identify biomarkers related to pCR. The cells were separately quantified in the tumor and stroma. By systematically analyzing the correlation between important radiomic features in the final model and the aforementioned biomarkers, this study aimed to expand our understanding of the potential mechanism underlying disease occurrence and development, providing a valuable theoretical reference for clinical diagnosis and treatment.

## Statistical analysis

Statistical analyses were performed using Python version 3.7.1. Regarding clinical baseline characteristics, continuous variables were expressed as mean ± standard deviation and compared using the independent-samples t-test or the Mann–Whitney U test. Ordinal variables were presented as median (first quartile, third quartile) and compared using the chi-square test. It is important to note that a few minimal clinical features, such as the baseline hematological index, were missing. These missing values were primarily imputed using the median value. For radiomics features with missing data, the machine learning algorithm KNNImputer was employed for imputation. In the context of pathological validation, the t-test was used to evaluate correlations between the screened radiomics features and PD-L1CPS, with *P* < 0.05 regarded as statistically significant. Pearson correlation analysis was conducted to evaluate the association between peritumoral subregion features and screened biomarkers, with |R| > 0.75 indicating a strong correlation. To evaluate the discriminatory ability of the models, ROC curves, calibration curves, and confusion matrix were utilized, and model performance was evaluated by calculating the AUC, accuracy, recall, precision, and F1 score to comprehensively and objectively evaluate the reliability and validity of this study.

## Results

### Patient characteristics

The demographic characteristics of the study cohort are listed in Table 1. Among the 212 patients included in the analysis, 56 individuals (26.4 %) achieved pCR. Significant differences were observed between the pCR and non-pCR groups in pre-N status (*P* = 0.042), number of immunotherapy cycles (*P* = 0.006), post-T status (*P* = 0.006), PLT (*P* = 0.019), and MON (*P* = 0.046).

**Table 1:** Patient characteristics

	pCR	Non-pCR	*P*
Sex			0.096
Female	6(10.7%)	7(4.5%)	
Male	50(89.3%)	149(95.5%)	
Smoking history			0.579
NO	14(25%)	29(18.6%)	
Yes	27(48.2%)	84(53.8%)	
Quit	15(26.8%)	43(27.6%)	
Drinking history			0.139
No	32(57.1%)	66(42.3%)	
Yes	16(28.6%)	54(34.6%)	
Quit	8(14.3%)	36(23.1%)	
preT			0.911
T1	1(1.8%)	1(0.6%)	
T2	6(10.7%)	13(8.3%)	
T3	19(33.9%)	57(36.5%)	
T4a	20(35.7%)	59(37.8%)	
T4b	10(17.9%)	26(16.7%)	
preN			0.042^a^
N0	24(42.9%)	54(34.6%)	
N1	8(14.3%)	24(15.4%)	
N2a	1(1.8%)	3(1.9%)	
N2b	5(8.9%)	43(27.6%)	
N2c	4(7.1%)	13(8.3%)	
N3a	0(0%)	0(0%)	
N3b	14(25%)	19(12.2%)	
preStage			0.231
II	1(1.8%)	6(3.8%)	
III	16(28.6%)	37(23.7%)	
IVa	19(33.9%)	75(48.1%)	
IVb	20(35.7%)	38(24.3%)	
Number of induction cycles			0.006^a^
1	3(5.4%)	2(1.3%)	
2	26(46.4%)	85(54.5%)	
3	23(41.1%)	58(37.2%)	
4	0(0%)	10(6.4%)	
5	4(7.1%)	1(0.6%)	
postT			0.006^a^
T0	8(14.3%)	23(14.7%)	
T1	4(7.1%)	19(12.2%)	
T2	6(10.7%)	38(24.4%)	
T3	28(50%)	35(22.4%)	
T4a	6(10.7%)	23(14.7%)	
T4b	4(7.1%)	18(11.5%)	
postN			0.702
N0	33(58.9%)	77(49.4%)	
N1	8(14.3%)	25(16%)	
N2a	1(1.8%)	5(3.2%)	
N2b	7(12.5%)	32(20.5%)	
N2c	2(3.6%)	7(4.5%)	
N3a	0(0%)	0(0%)	
N3b	5(8.9%)	10(6.4%)	
postStage			0.881
0	16(28.6%)	38(24.4%)	
I	7(12.5%)	23(14.7%)	
II	5(8.9%)	14(9.0%)	
III	13(23.2%)	31(19.9%)	
IVa	12(21.4%)	34(21.8%)	
IVb	3(5.4%)	16(10.3%)	
BMI	23.7 ± 4.4	24.1 ± 4.1	0.919
PLT	242.0 ± 78.5	244.2 ± 60.3	0.019^a^
AGE	54(46.2,59.0)	50(44.0,56.3)	0.146
WBC	6.9(5.8,8.4)	7.1 (6.0,8.2)	0.620
NEU	4.17(3.2,5.4)	4.34(3.5,5.2)	0.549
Monocyte value	0.49(0.4,0.6)	0.6(0.4,0.7)	0.135
MON	6.7(5.6,7.5)	7.2(5.9,8.4)	0.046^a^
HGB	150(137.8,160.3)	150(140.0,158.0)	0.906
PDW	11.6(10.8,13.3)	11.6(10.4,12.8)	0.577
ALT	23(16.9,33.0)	24.0(17.1,39.5)	0.458
AST	20.3(16.3,28.1)	21.45(17.4,29.0)	0.369
ALB	43.3(41.0,45.0)	43.2(41.1,45.5)	0.802
GLO	28.2 (25.7,32.4)	27.5(25.0,30.8)	0.214
AGR	1.5 (1.3,1.7)	1.6 (1.4,1.7)	0.250

^a^ Statistically significant. AGR: albumin-globulin ratio; ALB: albumin; ALT: alanine aminotransferase; AST: aspartate transaminase; BMI: body mass index; GLO: globulin; HGB: hemoglobin; MON: monocyte; NEU: neutropenia; PDW: platelet distribution width; PLT: platelet; WBC: white blood cell count.

### The comparison between different models

**Figure 3.**
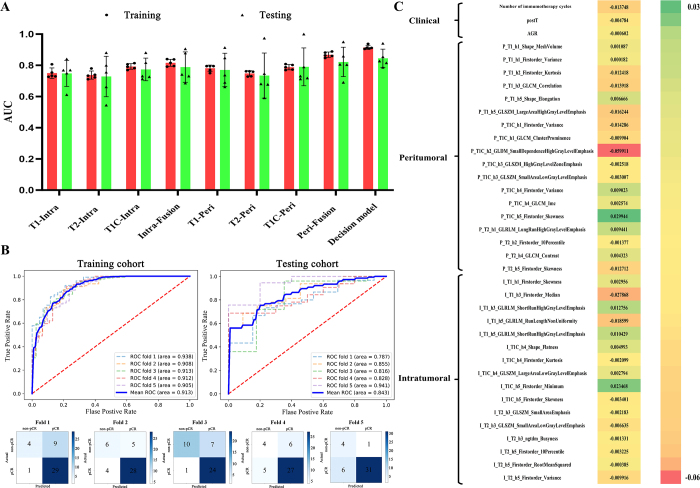
Comparison of different models and feature details in the decision model. (A) Box-and-whisker plots of AUC values for all models. (B) ROC curves in the training and testing cohorts for the decision model. AUC values are expressed as the mean and standard deviation across the five training and validation sets from the 5-fold cross-validation. The confusion matrices with heatmaps for the five validation sets of the decision model. The numbers in each colored box represent the percentage of instances between the true and the predicted classes obtained by the decision models. ROC, receiver-operating characteristic; AUC, area under the curve. (C) Details of clinical, peritumoral habitat features (PHF_S_) and intratumoral habitat features (IHF_S_) included in the decision model. T1, T1-weighted imaging; T1C, contrast-enhanced T1-weighted imaging with fat suppression; T2, axial T2-weighted imaging with fat suppression; Intra, intratumoral; Peri, peritumoral; P, peritumoral; I, intratumoral; h, habitat; GLCM, gray-level co-occurrence matrix; GLRLM, gray-level run-length matrix; GLSZM, gray-level size zone matrix; NGTDM, neighborhood gray-tone difference matrix.

Given that LR algorithm consistently outperformed other machine learning algorithms across different models (Supplemental Digital Content, Table S2, available at: http://links.lww.com/JS9/E436; Table S3, available at: http://links.lww.com/JS9/E437; Table S4, available at: http://links.lww.com/JS9/E438), this study primarily presents the results derived from LR. Figure 3A and Table 2 summarize the performance of the HI and Fusion models, with all LR algorithms achieving satisfactory results. The peritumoral HI and Fusion models were minimally higher than those of the intratumoral models. Specifically, peritumoral HI models demonstrated average AUC ranges of 0.748–0.788 (training) and 0.734–0.790 (testing), while peritumoral Fusion model achieved 0.869/0.822. In comparison, intratumoral HI models showed AUCs of 0.738–0.793 (training) and 0.729–0.774 (testing), and intratumoral Fusion model scored 0.817/0.789. Complementary performance metrics, including precision, recall, and F1 scores, also further supported these trends (Table 2). Specifically, peritumoral HI models demonstrated superior discriminative ability with precision ranging from 0.777–0.797 (training) and 0.759–0.782 (testing), recall values between 0.944–0.956 (training) and 0.901–0.928 (testing), and F1 scores of 0.855–0.869 (training) and 0.828–0.837 (testing). Intratumoral HI models achieved comparable results, with precision of 0.767–0.802 (training) and 0.752–0.770 (testing), recall of 0.930–0.966 (training) and 0.900–0.968 (testing), and F1 scores of 0.848–0.861 (training) and 0.819–0.852 (testing). Fusion models exhibited consistent improvements across these metrics. Peritumoral Fusion model attained precision/recall/F1 scores of 0.831/0.930/0.877 (training) and 0.783/0.902/0.827 (testing), while intratumoral Fusion model achieved 0.816/0.942/0.874 (training) and 0.803/0.926/0.789 (testing).

**Table 2:** Performance of the intratumoral and peritumoral habitat imaging (HI) models across different sequences with corresponding fusion models

	AUC	Accuracy	Recall	Precision	F1-score	PPV	NPV
T1-Intra							
Train	0.750	0.765	0.966	0.772	0.858	0.772	0.684
Test	0.748	0.759	0.968	0.770	0.852	0.770	0.640
T1C-Intra							
Train	0.793	0.781	0.930	0.802	0.861	0.802	0.656
Test	0.774	0.722	0.900	0.769	0.824	0.769	0.465
T2-Intra							
Train	0.738	0.738	0.948	0.767	0.848	0.767	0.585
Test	0.729	0.708	0.914	0.752	0.819	0.752	0.404
Intra-Fusion							
Train	0.817	0.801	0.942	0.816	0.874	0.816	0.715
Test	0.789	0.774	0.926	0.803	0.789	0.803	0.663
T1-Peri							
Train	0.781	0.789	0.956	0.797	0.869	0.797	0.733
Test	0.771	0.741	0.926	0.773	0.837	0.773	0.551
T1C-Peri							
Train	0.788	0.777	0.944	0.791	0.861	0.791	0.687
Test	0.790	0.736	0.901	0.782	0.828	0.782	0.550
T2-Peri							
Train	0.748	0.763	0.950	0.777	0.855	0.777	0.642
Test	0.734	0.731	0.928	0.759	0.831	0.759	0.490
Peri-Fusion							
Train	0.869	0.810	0.930	0.831	0.877	0.831	0.718
Test	0.822	0.727	0.902	0.783	0.827	0.783	0.493

AUC: area under curve; Intra: intratumoral; NPV: negative predictive value; Peri: peritumoral; PPV: positive predictive value; T1: T1-weighted imaging; T1C: contrast-enhanced T1-weighted imaging with fat suppression; T2: axial T2-weighted imaging with fat suppression.

An optimal decision model was constructed based on peritumoral habitat features (PHF_S_), intratumoral habitat features (IHF_S_), and clinical features. The average AUC values of the decision model in the training and testing sets were 0.913 and 0.843, respectively. In terms of precision, recall, and F1 score, the decision model achieved values of 0.884, 0.955, and 0.918 in the training set, whereas in the testing set these values were 0.835, 0.897, and 0.840. Details are presented in Table 3 and Fig. 3B. Figure 3C displays the selected features and their corresponding coefficients in this model, consisting of 18 PHF_S_, 16 IHF_S_, and 3 clinical features (number of induction cycles, postT, AGR). Meanwhile, Fig. 3B delineates the ROC curve of the decision model and the confusion matrix in the 5-fold cross-validation set. The calibration curves for this model in the training and testing sets are presented in Supplemental Digital Content, Figure S2 (available at: http://links.lww.com/JS9/E434).

**Table 3:** Performance of the decision model with five-fold cross-validation

Fold	Cohort	AUC	Accuracy	Recall	Precision	F1-score	PPV	NPV
Fold 1	Train	0.938	0.888	0.96	0.896	0.927	0.896	0.853
	Test	0.787	0.767	0.967	0.763	0.853	0.763	0.800
Fold 2	Train	0.908	0.852	0.952	0.861	0.904	0.861	0.812
	Test	0.855	0.791	0.875	0.848	0.812	0.848	0.600
Fold 3	Train	0.913	0.888	0.969	0.894	0.93	0.894	0.857
	Test	0.816	0.810	0.96	0.774	0.857	0.774	0.909
Fold 4	Train	0.912	0.888	0.96	0.895	0.926	0.895	0.865
	Test	0.816	0.81	0.96	0.774	0.857	0.774	0.909
Fold 5	Train	0.905	0.859	0.933	0.874	0.902	0.874	0.814
	Test	0.941	0.833	0.838	0.969	0.814	0.969	0.400
Mean	Train	0.913	0.875	0.955	0.884	0.918	0.884	0.840
	Test	0.843	0.788	0.897	0.835	0.840	0.835	0.631

AUC: area under curve; NPV: negative predictive value; PPV: positive predictive value.

### Interpretability analysis

**Figure 4.**
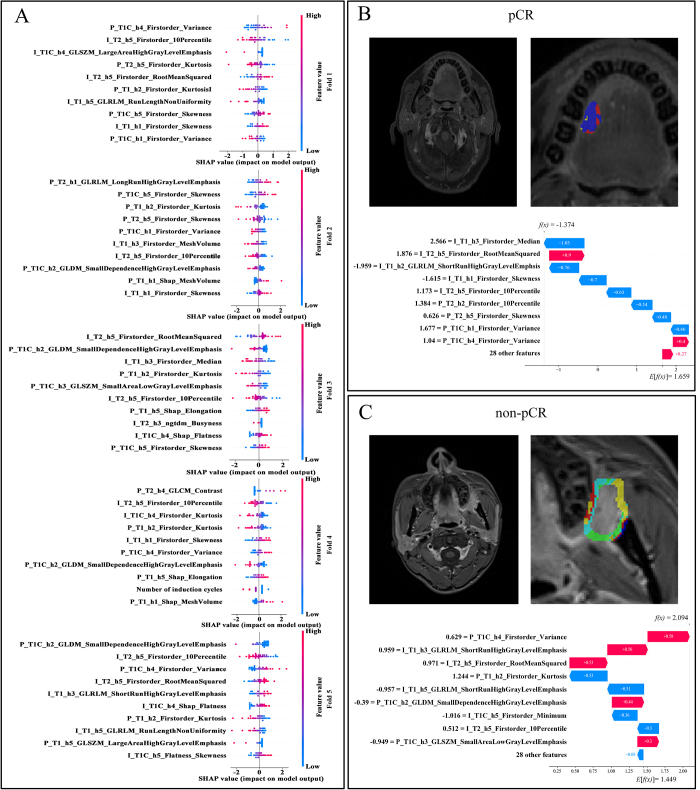
SHAP analysis in the decision model. (A) The SHAP bees-warm plot shows the position or negative effects of each feature on the prediction probability through rad and blue colors on a 5-fold training cohort. SHAP summary plot depicting the global prediction impact of features on the decision model and interaction between features. The importance of features is listed from top to bottom. Each point represents the SHAP value of a patient’s feature. Dots to the left of the Y-axis increase the likelihood of pCR, while dots to the right decrease the probability of pCR. SHAP, Shapley additive explanations. Patients (B) (pCR) and (C) (non-pCR) were randomly selected from the decision model. The waterfall plot shows that the impact of the ranking features on the model for a specific patient, where red represents a positive impact and blue represents a negative impact. P, peritumoral; I, intratumoral; h, habitat; GLCM, gray-level co-occurrence matrix, GLRLM, gray-level run-length matrix, GLSZM, gray-level size zone matrix, NGTDM, neighborhood gray-tone difference matrix; pCR, pathologic complete response.

SHAP analysis was conducted to rank the importance of features in the 5-fold cross-validation models to enhance the interpretability of the optimal decision model (Fig. 4A). Interestingly, the PHF_S_ were more dominant, considering that four of the top-ranked radiomics features in terms of importance were PHF_S_. Furthermore, one case was randomly selected from each of the pCR and non-pCR groups for illustrative purposes. The results revealed that within the pCR group, IHF was ranked highest in terms of importance (Fig. 4B). Conversely, in the non-pCR group, PHF held the top position (as depicted in Fig. 4C). Given the relatively comparable importance of certain features (as delineated in Supplemental Digital Content, Figure S3, available at: http://links.lww.com/JS9/E434), the top three features from each individual cross-validation model were selected for subsequent analysis. The specific features are summarized in Table 4.

**Table 4:** Associations between the key radiomic features selected by SHAP and both PD-L1 CPS and CD45+ in the stroma

Radiomics features	CPS		CD45+	
	t	*P*	r	*P*
P_T1_h2_Firstorder_Kurtosis	−0.357	0.723	0.406	0.423
P_T1C_h2_GLDM_SmallDependence HighGrayLevelEmphasis	−0.339	0.736	0.958	0.003^a^
P_T1C_h4_Firstorder_Variance	−0.649	0.521	−0.919	0.009^a^
P_T1C_h5_ Firstorder _Skewness	2.222	0.033^a^	−0.164	0.754
P_T2_h1_GLRLM_LongRunHigh GrayLevelEmphasis	−0.813	0.422	0.343	0.504
P_T2_h4_GLCM_Contrast	−0.304	0.762	−0.224	0.668
I_T1_h3_Firstorder_Median	2.344	0.025^a^	−0.173	0.742
I_T1C_h4_Firderorder_Kurtosis	0.201	0.841	−0.281	0.588
I_T1C_h4_GLSZM_LargeAreaLowGray LevelEmphasis	2.027	0.069	−0.102	0.846
I_T2_h5_Firstorder_10Percentile	2.275	0.029^a^	0.131	0.804
I_T2_h5_Firstorder_RootMean Squared	2.233	0.032^a^	0.065	0.901

h: habitat; I: intratumoral; P: peritumoral; T1: T1-weighted imaging; T1C: contrast-enhanced T1-weighted imaging with fat suppression; T2: axial T2-weighted imaging with fat suppression.

^a^ Statistically significant.

**Figure 5.**
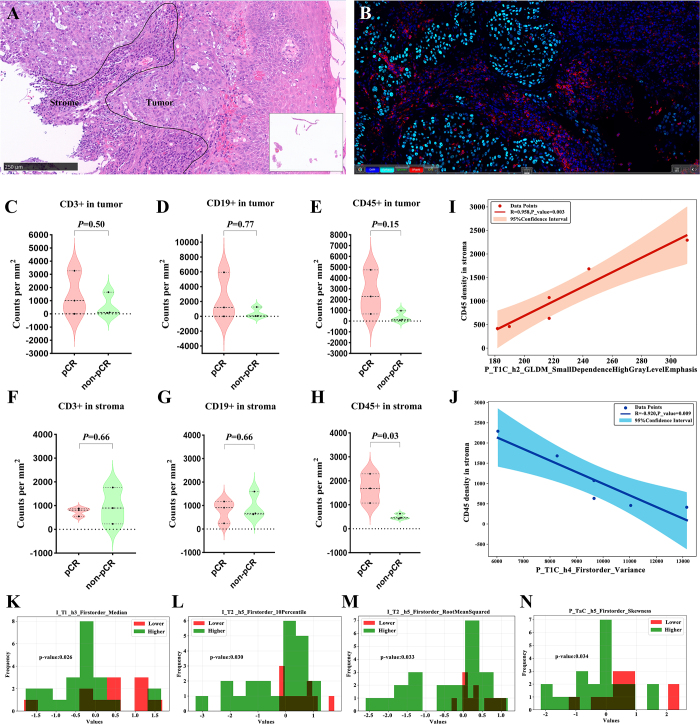
Biological Interpretability Analysis. (A) Pathological specimen section. (B) Hematoxylin–eosin (H&E) staining. (C–E) Comparison of CD3+, CD19+, and CD45+ in tumors between pCR and non-pCR groups. (F–H) Comparison of CD3+, CD19+, and CD45+ in stroma between pCR and non-pCR groups. (I) Correlation between P_T1C_h2_GLDM_SmallDependenceHighGrayLevelEmphasi and CD45+ in stroma. (J) Correlation between P_T1C_h4_Firstorder_Variance with CD45+ in stroma. (K–N) Distribution of radiomic features with statistical significance among patients stratified into different PD-L1 CPS groups. I_T1_h3_Firstorder_Median (K), I_T2_h5_ Firstorder_10Percentile (L), I_T2_h5_ Firstorder_RootMeanSquared (M), P_T1C_h5_ Firstorder_Skewness (N) . Intra, intratumoral; Peri, peritumoral; h, habitat; GLDM, gray-level dependence matrix; T1, T1-weighted imaging; T1C, contrast-enhanced T1-weighted imaging with fat suppression; T2, axial T2-weighted imaging with fat suppression; pCR, pathologic complete response.

Further biological interpretability analysis demonstrated that CD45+ white blood cell densities were significantly higher in the stroma of pCR patients compared to non-pCR patients (*P* = 0.03) (Fig. 5A-H). Moreover, two PHFs (P_T1C_h2_dm_SmallDependenceHighGrayLevelEmphasis, P_T1C_h4_F_Variance) among the aforementioned features were highly correlated with CD45+ white blood cell densities in the stroma (r = 0.958, −0.920, *P* < 0.05) (Table 4 and Fig 5I-J). In addition, this study preliminarily explored the relationship between the aforementioned features and PD-L1 CPS. Three IHFs (I_T1_h3_Firstorder_Median,I_T2_h5_Firstorder_10Percentile, I_T2_h5_Firstorder_RootMeanSquared) and one PHF (P_T1C_h5_Firstorder_Skewness) could distinguish between lower and higher CPS scores (t = 2.027 ~ 2.275, *P* < 0.05) (Table 4 and Fig 5K-N).

## Discussion

To the best of our knowledge, this is the first study to apply multi-parameter MRI-based intratumoral and peritumoral HI to predict pCR following NACI in OSCC patients. As anticipated, the overall efficacy of the peritumoral HI models was marginally higher than that of the intratumoral HI models. The decision model composed of 18 PHFs, 16 IHFs, and 3 clinical features (Number of induction cycles, postT, AGR) achieved the highest efficacy. In terms of biological verification, among the 11 most important features screened by SHAP, 3 ITHs and 1 PTHs were associated with PD-L1 CPS, whilst 2 PTHs were highly correlated with CD45+ white blood in the stroma, which was correlated with pCR.

Notably, traditional radiomics methods have been extensively applied in OSCC, yet research on predicting the pCR of NACI for OSCC remains extremely limited. Liu *et al*[14] extracted histogram features from pre- and post-treatment MRI images (T1WI, T2WI, T1C) and ADC maps of HNSCC patients. Their models demonstrated promising predictive ability (AUC = 0.83), which is lower than that of our models (AUCs = 0.913 and 0.843 in the training and test sets, respectively). Moreover, the sample size of their study was relatively small, with only 11 out of 24 patients achieving pCR. In another study, Lin *et al*[12] integrated intratumoral and peritumoral radiomics features derived from T2WI and T1C sequences to predict pCR in HNSCC, achieving high AUC values (0.904, 0.860 and 0.849 in the internal training, test and external validation sets). However, their final model included PD-L1 CPS, which necessitates invasive testing and expensive cost. In contrast, our model does not directly incorporate biomarkers. Instead, we identified intratumoral and peritumoral habitat radiomics features associated with biomarkers such as PD-L1 CPS and CD45+ cell, and used them to develop the decision model. This approach enables our predictive model to have broader applications.

Furthermore, the integration of ITFs and PTFs into our decision model demonstrated superior predictive performance for pCR in comparison to single intratumoral or peritumoral HI model. It is noteworthy that the peritumoral HI model exhibited a modest advantage over the intratumoral HI model. This result aligns with the findings of previous studies that investigated the prediction of epidermal growth factor receptor (EGFR) mutations[32], the invasiveness of lung adenocarcinoma[31], and microvascular invasion in hepatocellular carcinoma[33]. Intratumoral HI model can elucidate the internal biological behavior of tumors, the integrated model broadens the dimensionality of data by incorporating peritumoral features, enhancing its ability to distinguish between responders and non-responders to immunotherapy. The peritumoral region functions as a critical interface between tumor tissue and normal tissue, contain such as immune cells, blood vessels, fibroblasts, and the extracellular matrix. Research has demonstrated that cells within the peritumoral region, including lymphocytes, dendritic cells, high endothelial venule cells, and myofibroblastic cancer-associated fibroblasts, exert a substantial influence on the immune response to tumor cells[40]. Concurrently, peritumoral blood vessels, induced by factors such as vascular endothelial growth factor (VEGF), influence drug delivery and immune cell trafficking. Consequently, the intricate tumor microenvironment in the peritumoral area is instrumental in determining the efficacy of immunotherapy. These factors facilitate a more comprehensive reflection of the tumor’s biological behavior within the peritumoral region, consequently enhancing the performance of predicting pCR. This finding is consistent with the observations reported in previous research^[31-33]^.

In terms of the biological interpretability of imaging features, this study identified, through SHAP analysis, that two PHFs were highly correlated with CD45+ cells in the stroma, while multiple IHFs were associated with the PD-L1 CPS in tumor cells. CD45, a leukocyte marker, encompasses various immune cell subsets, including T lymphocytes, B lymphocytes, macrophages, dendritic cells, and neutrophils. Our findings indicate that CD45+ cells (i.e., leukocytes) in the peritumoral stroma prior to treatment are associated with pCR in OSCC. Higher CD45+ cell density has been demonstrated to be a significant predictor of enhanced immune-tumor interactions and improved responses to immunotherapy in this study. Meanwhile, these PHFs may potentially serve as indicators for monitoring changes in the peritumoral immune microenvironment, providing a novel perspective for evaluating the efficacy of immunotherapy. Numerous studies had demonstrated a positive correlation between higher levels of immune cells, particularly specific CD8+ T cell subsets, and enhanced survival outcomes^[41,42]^. Conversely, the PD-L1 CPS in tumor cells functions as a significant biomarker for predicting the efficacy of immunotherapy in head and neck cancer. Patients with higher CPS scores frequently demonstrate superior immune responses[43]. Also, there are some studies that investigated the microenvironment of HNSCC through the application of radiomics. For instance, Wang *et al*[44].employed preoperative CT-based radiomics to predict the enrichment status of CD8+ T cells in HNSCC. Katsoulakis *et al*[4] developed a model to predict CD8+ T cell infiltration in HNSCC through radiomic analysis using data from The Cancer Imaging Archive and The Cancer Genome Atlas. In the present study, IHFs were found to be predominant in a randomly selected patient sample with pCR, and multiple IHFs were correlated with CPS. In a randomly selected non-pCR patient sample, PHFs were found to be predominant. It is imperative that future research endeavors prioritize the execution of expansive sample studies, which are essential for further exploration and elucidation of the underlying phenomena.

Nevertheless, this study is subject to several limitations. Firstly, as a retrospective study, there are risks of selection and recall bias. Despite the employment of a medium-sized sample, five-fold cross-validation, and stable radiomics features to reduce overfitting[45], biases may still exist. Furthermore, the performance of the model may be subject to confounding by multiple clinical covariates, including tumor localization and chemotherapy strategy. Therefore, a prospective, multi-center study is required. Secondly, although correlations between tumor microenvironment radiomics features and PD-L1 CPS, CD45+ cells in the stroma were established, further immunological validation at the tissue and molecular level is required. Thirdly, in order to minimize data consistency discrepancies across different centers, the use of functional MRI image such as diffusion weighted imaging (DWI) was excluded. Subsequent research will investigate multi-sequence combinations to enhance prediction accuracy. Finally, in the absence of a consensus definition of the peritumoral region^[32,33,46]^, it is acknowledged that variations in the definition may have a consequential impact on the performance of the models. Further study was need to conduct an in-depth exploration of the effects of different peritumoral areas on radiomics models, and also investigate the associations between the radiomics features and biomarkers to determine the optimal definition of the peritumoral region.

In summary, the MR-based integrated intratumoral and peritumoral HI models can provide valuable information for predicting the response to NACI in OSCC, and SHAP interpretability analysis enhances the interpretability of the decision model, thereby enabling the formulation of personalized treatment for patients.

## Data Availability

All data generated for this study are available from the corresponding author upon reasonable request.

## Research registration unique identifying number (UIN)

NCT06854107. https://www.clinicaltrials.gov/study/NCT06854107?cond=NCT06854107&rank=1.
